# Feigned Hysteria

**Published:** 1842-03

**Authors:** 


*Feigned Hysteria.*—Some of the shapes assumed by this pathological proteus are hideous and disgusting. Paralysis of the muscular fibres of the bladder, or spasm of its sphincters, sometimes really occurs; sometimes it is only aped, in hysteria. It is a common trick with these patients to pretend that they labor under *retention of urine*, and that, although the bladder is full, they cannot make water. The daily introduction of the catheter by a dresser or apprentice, appears to gratify their morbid or prurient feelings. Sometimes, no doubt, the difficulty is real, but it is oftener feigned or exaggerated. I have again and again known it to disappear, upon the patient's being left, without pity, to her own resources. But girls have been known to drink their urine, in order to conceal the fact of their having been obliged and able to void it. The state of mind evinced by many of these hysterical young persons, is such as to entitle them to our deepest commiseration. The deceptive appearances displayed in the bodily functions and feelings find their counterpart in the mental. The patients are deceitful, perverse and obstinate, practising, or attempting to practice, the most aimless and unnatural impositions. They will produce fragments of common gravel, and assert that these were voided with the urine, or they will secrete cinders and stones in the vagina, and pretend to be suffering under some calculous disease. A young woman contrived, in one of our hospitals, to make the surgeons believe that she had *stone in the bladder*, and she actually submitted to be placed on the operating table, and to be tied up in the posture for lithotomy, before a theatre full of students, and then the imposture was detected. Sometimes they simulate *suppression of urine*, and after swallowing what they have passed, vomit it up again, to induce the belief that the secretion has taken place through a new and unnatural channel.

It is impossible, I say, not to pity the unhappy victims of this wretched disorder, when their morbid propensities drive them to such acts as these. I mention them, because you must expect to meet with such cases, and because, while you take care not to express your suspicions prematurely, or on light evidence, you should be on your guard against the mortification of being deceived, by the false signals held out, into active and ill directed measures of treatment.—*Dr. Watson's Lectures on the Practice of Medicine*, in *Lond. Med. Gazette*, June 11, 1841.

